# Impact of an Innovative Securement Dressing and Tourniquet in Peripheral Intravenous Catheter-Related Complications and Contamination: An Interventional Study

**DOI:** 10.3390/ijerph16183301

**Published:** 2019-09-08

**Authors:** Pedro Parreira, Beatriz Serambeque, Paulo S. Costa, Lisete S. Mónico, Vânia Oliveira, Liliana B. Sousa, Fernando Gama, Rafael A. Bernardes, David Adriano, Inês A. Marques, Luciene M. Braga, João Graveto, Nádia Osório, Anabela Salgueiro-Oliveira

**Affiliations:** 1 Health Sciences Research Unit: Nursing (UICISA: E), Nursing School of Coimbra (ESEnfC), 3000-232 Coimbra, Portugal (P.P.) (B.S.) (P.S.C.) (L.B.S.) (I.A.M.) (J.G.) (A.S.-O.); 2 IPCDVS, Faculty of Psychology and Education Sciences, University of Coimbra, 3000-115 Coimbra, Portugal; 3 Institute for Biomedicine-iBiMED, University of Aveiro, 3810-193 Aveiro, Portugal; 4 Coimbra Hospital and Universitary Centre (CHUC), 3004-561 Coimbra, Portugal (F.G.) (D.A.); 5 Nursing Department, Federal University of Viçosa, Viçosa 36570-900, Brazil; 6 Department of Biomedical Laboratory Sciences, Coimbra Health School (ESTeSC), Polytechnic Institute of Coimbra, 3046-854 Coimbra, Portugal

**Keywords:** catheter-related bloodstream infections, complications, contamination, infection prevention, nursing, occlusive dressings, peripheral intravenous catheter, tourniquets

## Abstract

Reusable tourniquets and conventional securement dressings are considered risk factors for the occurrence of reported complications and catheter-related bloodstream infections. This study’s purpose is to assess the impact of single-use disposable tourniquets and advanced occlusive polyurethane dressings with reinforced cloth borders on peripheral intravenous catheter (PIVC)-related complications and contamination. A pre- and post-interventional prospective observational study was conducted in a cardiology ward of a tertiary hospital between April 2018 and February 2019. Overall, demographic and clinical data from 156 patients and PIVC-related outcomes were collected (*n* = 296) as well as PIVC tips for microbiological analysis (*n* = 90). In the pre-intervention phase (*n* = 118), complication rates of 62.1% were reported, while 44.1% of the PIVCs were contaminated (*n* = 34). In the post-intervention phase (*n* = 178), complication rates decreased to 57.3%, while contamination rates significantly decreased to 17.9% (*p* = 0.014; *n* = 56). Through a logistic regression, it was found that the use of innovative technologies reduces the chance of PIVC contamination by 79% (odds ratio (OR): 0.21; 95% confidence interval (CI): 0.05–0.98; *p* = 0.046). Meanwhile, PIVC-related complications and fluid therapy emerged as predictors for PIVC contamination. Findings suggest that the adoption of these innovative devices in nurses’ practice contributes to the significant reduction of PIVC contamination.

## 1. Introduction

Peripheral intravenous catheter (PIVC) placement is an invasive procedure, often performed in patients admitted to the clinical setting [1,2,3]. Most inpatients require at least one PIVC during their hospital admission; however, up to 69% of PIVCs fail and require premature removal [4,5,6,7,8]. The premature failure of PIVCs and the risk of developing local and systemic complications associated with their insertion and maintenance can result in more extended hospital stays and distress for patients [9,10,11]. The most frequently reported PIVC-related complications are phlebitis, infiltration, obstruction, extravasation, and accidental removal [3,4,8,12,13]. However, catheter-related bloodstream infections are also highlighted in contemporary literature as a potential negative PIVC outcome [14,15,16].

Among the different factors that may contribute to the occurrence of infection, reusable tourniquets are considered a risk factor for the development of puncture site infections due to the lack of references on correct decontamination procedures between patients [17,18,19]. Current international guidelines recommend the use of single-use disposable tourniquets to reduce the incidence of infections associated with their usage [20,21]. To our knowledge, there is only one study reporting the use of single-use disposable tourniquets, yet the assessment of PIVC contamination after its implementation was not performed [19].

PIVC-related complications can also be avoided by providing cutaneous antisepsis care [22]. Moreover, adequate PIVC securement and puncture site protection with a sterile dressing is recommended in order to lower the risk of PIVC-related contamination [20,21,23]. Nevertheless, there is no evidence of which type of dressing is the most effective in the prevention of PIVC failure [1].

The purpose of this study is to assess the impact of single-use disposable tourniquets and occlusive dressings with reinforced borders on PIVC-related complications and contamination.

## 2. Materials and Methods 

### 2.1. Study Design and Setting

An action-research project was implemented in a cardiology ward of a tertiary hospital in central Portugal, with the involvement of the nursing team in the research process. In the selected setting, PIVCs are inserted by the nursing team after indication by the medical team. Vein selection is performed through the palpation and observation of anatomical landmarks, without the assistance of common vein selection technologies (e.g., ultrasound or near-infrared light). PIVC maintenance is performed daily by the nursing team through the visual inspection of the dressing integrity and insertion site. PIVCs are removed by the nursing team when clinically indicated (e.g., end of intravenous treatment) or replaced when complication signs/symptoms are found.

In a pre-intervention phase, between 9 April 2018 and 31 August 2018, a prospective observational study was developed. The ward nurses recorded the professional practices and the technologies used in peripheral venous catheterization, as well as PIVC-related complications. In this phase, traditional rubber or textile reusable tourniquets and conventional securement transparent dressings were used during PIVC insertion and maintenance. When removed, catheter tips were sent for microbiological analysis, provided that they were collected during the laboratory’s working period (8:00 a.m. to 3:00 p.m.). Two researchers, responsible for sending samples to the laboratory, remained in the care unit at the time of PIVC tips’ collection to ensure that the data procedures were strictly carried out. PIVC contamination was evaluated through the semiquantitative technique proposed by Maki and associates [24].

In the intervention phase, two workshops of discussion/reflection were conducted among the nursing and research teams on 19 and 26 September 2018. In each workshop, in the first round, the researchers’ team presented the key points of the data collected during the pre-intervention phase and discussed them in light of recent scientific evidence. In the second round, innovative technologies (single-use disposable tourniquets and occlusive dressings with reinforced borders) were also presented as an alternative to those used in the care unit for the prevention of PIVC-related infections.

In a post-intervention phase, between 28 September 2018 and 28 February 2019, the same care protocol was followed; however, nurses adopted in their clinical practice single-use disposable tourniquets and occlusive dressings with reinforced borders. The single-use disposable tourniquets are made of a special paper and plastic composite material, and the occlusive dressings with reinforced borders are 7 × 8 cm advanced polyurethane dressings with adhesive cloth borders. In addition, if the dressings remain intact without leakage, they protect the insertion site from external contamination. Following the same method employed in the pre-interventional phase, PIVC tips were also collected for microbiological analysis. Likewise, the ward nurses recorded data related to PIVC insertion, maintenance, and removal daily.

In the pre- and post-interventional phases, data were collected using a well-structured form developed by the research team, based on recent international standards of care and guidelines [20,21,23]. The variables recorded focused on patients’ demographic and clinical status, PIVC-insertion related outcomes (selected puncture site, catheter gauge size, number of puncture attempts until success, easiness of puncture (using a 7-point scale: 1–3, easy; 4, intermediate; 5–7, extremely difficult), number of simultaneous catheters, catheter dwell time, catheter fixation method, material of the used tourniquet, and antiseptic used). If the reason for PIVC removal was a complication, signs and symptoms were reported by the ward nurses for posterior classification as phlebitis, infiltration, obstruction, or accidental removal. To assist nurses in reporting such signs and symptoms, the Phlebitis Scale [25] and Infiltration Scale [26], culturally adapted and validated for the Portuguese population, were used after formal authorization was obtained from the authors. The Phlebitis Scale is an instrument graduated from zero (absence of phlebitis) to four (evident signs of thrombophlebitis), containing at each level the signs and/or symptoms of phlebitis and assisting professionals to determine the need to replace the PIVC [25]. The Portuguese version has two levels of severity with a Cronbach’s alpha of 0.78 (level one) and 0.90 (level two) [25]. The Infiltration Scale is organized into four levels, between zero (absence of infiltration) and four (severe infiltration), using clinical criteria to evaluate each level of infiltration, such as skin color, skin temperature to touch, pain, extent, and depth of edema [26]. The Portuguese version has a Cronbach’s alpha of 0.85 [26]. The description of the observational studies followed the Strengthening the Reporting of Observational Studies in Epidemiology (STROBE) Statement guidelines.

### 2.2. Participants

Eligible participants for both prospective observational studies were cardiology ward inpatients, 18 years of age or older, whose PIVCs were inserted by the nursing team during their stay. Patients who could not understand and communicate with the nursing/research team or who did not consent to their participation were excluded from the study.

In the post-intervention phase, the same inclusion and exclusion criteria were maintained; however, single-use disposable tourniquets and occlusive dressings with reinforced borders were implemented and used as part of the ward care protocol. All inserted PIVCs of each patient were included in the study.

The study sample consisted of all patients admitted to the cardiology ward who required PIVCs during the period established for each phase, being considered a convenience sample.

The nursing team of the cardiology ward also acted as participants, and their characterization was described in a previous work [27].

### 2.3. Ethical Considerations

This study was conducted in accordance with the Declaration of Helsinki of 1975 and was approved by the institution’s Ethics Committee (number 115-17) and by the Portuguese Data Protection Authority (authorization number 14037/2017). Throughout the study, all ethical considerations were strictly complied with, including informed consent and voluntary participation, ensuring all legal aspects regarding privacy and confidentiality. Patients with eligibility criteria received information about the study, and each patient or his/her legal representative delivered the informed consent for the study. As part of the action-research project, informed consent was also obtained from the members of the nursing team.

### 2.4. Data Analysis

Data analysis was performed using SPSS Statistics^®^ (version 24, IBM SPSS; Chicago, IL, USA). Means, standard deviations, frequencies, and percentages were used as descriptive statistics. Comparison between study groups (pre- and post-intervention) for demographic and clinical variables, as well as for the occurrence of complications and contamination of PIVCs, was assessed using the Chi-square, Fisher’s exact, and Student’s *t* tests. A significance level of 0.05 was considered significant. Regarding the incidence of PIVC contamination, catheters that presented 15 or more colony-forming units (CFUs), using the Maki technique, were considered contaminated with a potential risk to trigger a bloodstream infection [24].

A multivariate analysis was performed using the binary logistic regression model, Wald test, and odds ratio, along with the 95% confidence intervals. To define the variables that would integrate the regression model, a correlation was performed between the dependent variable, PIVC contamination, and all variables that presented a statistically significant correlation at *p* < 0.100.

## 3. Results

### 3.1. Patients’ Characterization

During the study’s implementation period, 193 inpatients were enrolled, but only 156 patients presented eligibility criteria; 37 patients were excluded due to difficulties in obtaining consent from the patient or his/her legal representative. Demographic and clinical status variables of eligible patients were similar between the pre- and post-intervention phases (Table 1). However, patient groups were not similar concerning intravenous therapeutics (χ^2^ (2) = 6.43; *p* = 0.040) and antibiotics (Fisher’s exact test; *p* = 0.028) because, in the pre-intervention phase, a higher number of patients was receiving intravenous (IV) therapy, especially antibiotics. 

### 3.2. Peripheral Intravenous Catheterization Characterization

In this study, 309 PIVC insertions were observed and recorded; 13 were excluded in the post-intervention phase (the innovative tourniquet and securement dressing were not used). One hundred and eighteen IV catheters were observed in the pre-intervention phase and 178 in the post-intervention phase. The description of the observed peripheral IV catheterizations in both phases is presented in Table 2. The pre- and post-intervention patient groups are not comparable regarding easiness of puncture (Student’s *t*-test: *p* = 0.005) and antiseptic used (Fisher’s exact test: *p* < 0.001).

In the pre-intervention phase, textile and rubber reusable tourniquets were used in 39% and 60.2% of the catheterizations, respectively. In one catheterization (0.8%), disposable gloves were used. Concerning dressing securement, transparent dressings were used in 98.3% of the PIVCs and transparent dressings overlapping with adhesive tape in 1.7% of the cases.

### 3.3. PIVC-Related Complications and Contamination

From the pre-intervention phase to the post-intervention phase, it was verified that the PIVC-related complications decreased, although the difference is not statistically significant, globally and for each of the complications presented (Table 3).

Regarding PIVC contamination, 90 catheters were sampled and analyzed, 34 and 56 in the pre- and post-intervention phases, respectively. There was a statistically significant decrease in PIVC contamination from pre- to post-intervention (Fisher’s exact test; *p* = 0.014).

Considering the significant decrease in PIVC contamination rate in the post-intervention phase, we tried to find out which variables could be contributing to this decrease. Regarding the demographic and clinical-related variables, it was observed that the groups are equivalent, except for the IV therapeutic and IV antibiotics. In relation to the PIVC insertion-related variables and catheter dwell time, the groups are not equivalent regarding the easiness of puncture and the used antiseptic. Therefore, the potential association between such variables and PIVC contamination was explored through a univariate analysis (Table 4). However, no statistically significant relationship was found.

Given the absence of statistical association between PIVC contamination and unequaled variables in pre- and post-intervention, a post-hoc analysis was performed to ascertain if the single-use disposable tourniquet and occlusive dressing with reinforced borders contributed to the decreasing of PIVC contamination, which were integrated into the analysis as a single variable (innovative tourniquet and dressing), since their implementation in clinical practice was simultaneous. In this analysis, 77 patients were included whose PIVC tips were microbiologically analyzed from the total patient sample of the study (n = 156). All variables under study were integrated into the correlation analysis.

Table 5 shows the correlations between PIVC contamination and statistically significant variables for *p* < 0.100.

As can be seen from Table 5, for a maximum type I error of 0.10, negative correlations can be found between PIVC contamination and the use of single-use disposable tourniquets and occlusive dressings with reinforced borders (*p* < 0.010), and also the use of chlorhexidine (*p* < 0.01), indicating some protection against PIVC contamination. Inversely, positive correlations were found between PIVC contamination and the number of PIVCs during hospital stay (*p* < 0.050), cumulative days with PIVCs (*p* < 0.050), fluid therapy (*p* < 0.010), and the presence of complications (*p* < 0.050).

A binary logistic regression was performed to ascertain if PIVC contamination was significantly predicted by single-use disposable tourniquets and occlusive dressings with reinforced borders, chlorhexidine, number of PIVCs during hospital stay, cumulative days with PIVCs, fluid therapy, and the presence of complications. Number of PIVCs during hospital stay and cumulative days with PIVCs were excluded from the analysis for non-significant predictive effects (*p* ≥ 0.66). 

The final logistic regression model was composed of the following predictors: single-use disposable tourniquets and occlusive dressings with reinforced borders, chlorhexidine, fluid therapy, and the presence of complications. It correctly classifies 75.7% of cases, being statistically significant according to the Omnibus tests of model coefficients, *G*^2^ (4) = 21.38, *p* < 0.001; and pseudo-R-squared *R*^2^*_CS_* = 0.236 and *R*^2^*_NN_* = 0.367. According to the Hosmer and Lemeshow test, predicted and observed values were statistically coincident, *X*^2^ (7) = 4.63, *p* = 0.705. The model presents 89.4% specificity to predict PIVC contamination, indicating that the model correctly classifies 89.4% of patients without PIVC contamination. The model has the sensitivity to predict PIVC contamination in 47.8% of cases. 

The regression coefficients of the adjusted logit model (see Table 6) were statistically significant for all predictors (*p* ≤ 0.058), excluding chlorhexidine (*p* = 0.302). The single-use disposable tourniquets and occlusive dressings with reinforced borders was a negative predictor, indicating a 79% lower chance of catheter contamination (OR = 0.21). PIVC contamination was highly increased by complications (estimated increase of 280% from the reference class 1 of risk of PIVC contamination, OR = 3.80), and also by fluid therapy (estimated increase of 226%, OR = 3.26). Chlorhexidine showed a tendency for a negative prediction of PIVC contamination (predicted decrease of 57%, OR = 0.43), although it was statistically non-significant in our data.

## 4. Discussion

The purpose of this action-research approach was to identify how the technologies adopted by nurses contributed to the reduction of PIVC-related complications and contamination. In this study, a decrease in peripheral venous catheterization-related complications (61.2% to 57.3%) was observed, whose clinical impact, although not statistically significant, is arguable. 

Marsh et al. (2015) [6] compared the efficacy of traditional polyurethane dressings with that of occlusive dressings with reinforced borders in PIVC securement and identified that traditional dressings are associated with higher catheter failure rates (38.1% versus 25%). However, in this study, the implementation of occlusive dressings with reinforced borders did not decrease PIVC-related complication rates between the pre- and post-interventional phases, which mirrors past results obtained by Rickard et al. (2018) [8]. The divergent results reported among recent studies highlight that the effectiveness of these last-generation dressings has not been thoroughly studied. However, our study introduces a potentially new research focus by comparing the impact that last-generation dressings have on PIVC contamination.

Overall, in both phases of this study, the occurrence of PIVC-related complications was significantly associated with several variables. Therefore, one cannot affirm that the implementation of innovative technologies between the pre- and post-interventional phases contributed per se to the reduction of complications.

Regarding PIVC contamination, in the pre-intervention phase, 44.1% of the analyzed PIVCs presented 15 or more CFUs, a similar result to that found by Pujol et al. (2007) [28] with 51.3%. 

However, in the post-intervention phase, PIVC contamination rates significantly decreased to 17.9%. In fact, the binary logistic regression analysis confirmed that innovative technologies are negative predictors of PIVC contamination because patients using the innovative tourniquet and dressing presented a 79% lower chance of the occurrence of PIVC contamination.

In the pre-intervention phase, conventional rubber or textile tourniquets used in peripheral IV catheterization constitute an optimal environment for pathogenic microorganisms’ growth, which can trigger bloodstream infections if professional practices and decontamination procedures are not followed correctly [27,29,30]. A descriptive-correlational study on the tourniquets used by nurses showed a contamination rate of 70.6% in non-disposable tourniquets used several times among patients [18]. A cross-sectional study found that 72.2% of the analyzed tourniquets were contaminated, and some of the microorganisms were resistant to antibiotic therapy [17]. However, if health professionals followed the guidelines recommended for the need to use single-use tourniquets during venous catheterization, it would lead to a reduction in the contamination associated with this practice [20,21]. Also, at this phase of the study, unreinforced transparent dressings were used and were often replaced to maintain puncture site protection and adequate catheter safety. A study by Timsit (2012) and colleagues showed that the number of dressing disruptions was associated with an increased risk of skin colonization at the puncture site, so such disruptions have been considered an important risk factor for the development of catheter-related infections [31]. In the post-intervention phase, the implementation of bordered polyurethane dressings was intended to provide greater strength through adhesive cloth borders [32]. 

However, from the analysis performed, there were predictors for the occurrence of PIVC contamination, namely the presence of PIVC-related complications and fluid therapy, which should be valued.

Regarding complications, patients with phlebitis, infiltration, obstruction or other individual signs, such as bleeding at the puncture site, presented a 280% higher chance of the occurrence of PIVC contamination. Nevertheless, the sample of our study did not allow the establishment of a relationship between contamination and each of the individual complications. In fact, injury to the vein endothelium during venous catheterization increases the risk of developing phlebitis and also infection [33,34]. Several authors evidenced that phlebitis is a favorable factor for bacterial colonization, allowing local infections, bloodstream infections and sepsis [9,35,36]. However, the scientific research found does not establish a relationship between the existence of other complications, such as infiltration or obstruction, and the development of contamination or PIVC-related infections.

Although IV therapy did not show a statistically significant correlation with PIVC contamination, the logistic regression analysis reveals that fluid therapy is a positive predictor, whose patients had a 226% higher chance of the occurrence of PIVC contamination. Corroborating this result, a study verified through a univariate analysis that the infusion of a hydroelectrolytic product presented a statistically significant relation with the occurrence of PIVC-related adverse effects, including infection [37]. 

In fact, we recorded the type of medication that each patient received during the study period, although we did not take into account the specifications of each therapy, namely whether fluid therapy was given on a continuous or intermittent approach. Despite this, the manipulation associated with the need for replacement of infusions and, if necessary, simultaneous administration of another medication, may contribute to an increase in PIVC contamination. Current international guidelines highlight that the recurrent manipulation of administration sets potentiate contamination and infection risks [21,38].

Regarding the antiseptic used during peripheral venous catheterization (alcohol/chlorhexidine), logistic regression analysis showed that chlorhexidine is a negative predictor, which reduces by 57% the chance of PIVC contamination, although without statistical significance. This result should not be neglected, since the guidelines suggest that chlorhexidine is recommended for the prevention of contamination [20,21].

Finally, the importance of the discussion and reflection panels conducted among the nursing and research teams during the intervention phase cannot be overlooked. Likewise, the constant involvement and openness of the nursing team toward the adoption of innovative technologies in their clinical practice were determinant. These actions were essential and in line with recommendations of recurrent health professionals’ education and training based on evidence-based recommendations for PIVC management [20,21].

To the best of the authors’ knowledge, this is the first study that focuses on the efficacy of single-use disposable tourniquets in reducing PIVC contamination. While our findings indicate that the use of single-use disposable tourniquets contributes to the reduction of PIVC contamination rates, more studies in this field are necessary. Given the likely impact that health professionals’ practices have on PIVC contamination when handling tourniquets [19,30], future studies on single-use disposable tourniquets should also attend to this aspect.

Overall, the results outlined must be analyzed within some limitations. The study groups are not comparable in some of the assessed patient-related characteristics and catheterization-related parameters (e.g., easiness of puncture, antiseptic used and reasons for PIVC removal). Despite the absence of a statistically significant relationship between PIVC contamination and unequaled variables in pre and post-intervention, the results obtained in this study constitute a valid and innovative contribution in the field. Nonetheless, future studies with larger sample sizes and randomized groups are needed to properly assess the impact of single-use disposable tourniquets and occlusive dressings with reinforced borders on PIVC-related complications and contamination, and in future studies, the antiseptic use should also be controlled. Future studies should also consider the impact of individual complications on PIVC contamination, as well as control the specifications of all administered IV therapies.

Regarding the assessment of PIVC contamination, in order to further explore this topic, future studies may include complementary microbiological analyses, namely blood cultures and swabs of the single-used disposable tourniquets (before patient contact) and puncture sites (during PIVC insertion and after skin antisepsis).

## 5. Conclusions

In this exploratory study, the implementation of innovative technologies in nurses’ practices contributed to the reduction of PIVC contamination rates, which can ultimately constitute a source of bloodstream infections. PIVC-related complications and fluid therapy emerged as variables that increase the chance of PIVC contamination; therefore, international recommendations and intituitions’ protocols should be followed by health professionals in order to prevent contamination.

Nonetheless, the implementation of such innovative technologies is not effective by itself, requiring the continuous involvement of health professionals as well as access to institutional educational and training actions based on the latest scientific evidence.

## Figures and Tables

**Table 1:** Demographic and clinical characteristics of study patients in each phase and the statistical significance between the study groups (*n* = 156).

Demographic and Clinical Characteristics	Pre-Intervention Phase (*n* = 54) *n* (%)	Post-Intervention Phase (*n* = 102) *n* (%)	*p* Value
Age * (years)	74.2 ± 14.3 (32–99)	75.9 ± 11.5 (40–101)	0.424 ^c^
Sex			
Female	23 (42.6)	40 (39.2)	0.733 ^b^
Male	31 (57.4)	62 (60.8)	
Comorbidities			
Diabetes mellitus	3 (6.3)	19 (18.6)	0.050 ^b^
Arterial hypertension	14 (29.2)	26 (25.5)	0.694 ^b^
Chronic kidney disease	4 (8.3)	8 (7.8)	1.000 ^b^
Missing	6	-	
Current infection	7 (14.6)	7 (6.9)	0.145 ^b^
Missing	6	1	
Number of peripheral intravenous catheters (PIVCs) during hospital stay *	2.2 ± 1.5 (1–7)	1.7 ± 1.5 (1–11)	0.103 ^c^
Cumulative days with PIVCs *	10.5 ± 8.7 (1–39)	9 ± 9.9 (0–72)	0.349 ^c^
Missing	-	3	
Intravenous (IV) therapeutics	42 (91.3)	87 (85.3)	0.040 ^a^
Missing	8	-	
IV antibiotics	24 (52.2)	33 (32.4)	0.028 ^b^
Missing	8	-	
Cardiovascular medication	23 (50.0)	58 (56.9)	0.478 ^b^
Noradrenaline	4 (8.7)	4 (3.9)	
Dobutamine	1 (2.2)	3 (2.9)	
Amiodarone	-	5 (4.9)	
Isosorbide dinitrate	1 (2.2)	5 (4.9)	
Missing	8	-	
Fluid therapy	18 (39.1)	48 (47.1)	0.475 ^b^
Missing	8	-	

* Mean ± SD (Min–Max), ^a^ Chi-square test, ^b^ Fisher’s exact test, ^c^ Student’s *t*-test, – No cases.

**Table 2:** Characterization of PIVC insertion and catheter dwell time in each phase and the statistical significance between pre- and post-intervention phases (*n* = 296).

PIVC-Related Variables	Pre-Intervention (*n* = 118)		Post-Intervention (*n* = 178)		*p* Value
	*n*	%	*n*	%	
Other inserted catheters					0.767 ^c^
None	97	82.2	143	82.2	
1 PIVC	18	15.3	28	16.1	
2 or more PIVC	3	2.5	3	1.7	
Total	118	100	174	100	
Missing	-		4		
Catheter site					0.922 ^a^
Hand	21	17.8	36	20.7	
Forearm	70	59.3	102	58.6	
Arm flexure	13	11.0	18	10.3	
Arm	14	11.9	18	10.3	
Total	118	100	174	100	
Missing	-		4		
Catheter gauge					0.210 ^a^
≤18 G	4	3.4	7	4.0	
20–22 G	114	96.6	168	95.0	
>22 G	0	0.0	2	1.1	
Total	118	100	177	100	
Missing	-		1		
Antiseptic solution					0.000 ^b^
70% Alcohol	80	67.8	28	15.7	
2% Alcoholic chlorhexidine	38	32.2	150	84.3	
Total	118	100	178	100	
Easiness of puncture	3.3 ± 1.9 (1–7)		2.7 ± 1.7 (1–7)		0.005 ^c^
Missing	-		3		
Number of attempts					0.358 ^c^
1	92	80.7	147	85.5	
2	15	13.2	18	10.5	
≥3	7	6.2	7	4.1	
Total	114	100	172	100	
Missing	4		6		
Catheter dwell time *	4.9 ± 3.6 (0–16)		5.1 ± 4.9 (0–26)		0.619 ^c^

* Mean ± SD (Min–Max), ^a^ Chi-square test, ^b^ Fisher’s exact test, ^c^ Student’s *t*-test, – No cases.

**Table 3:** Occurrence of complications associated with peripheral IV catheterization and the incidence of PIVC contamination in each phase along with the statistical significance between pre- and post-intervention phases.

PIVC-Related Complications and Contamination	Pre-Intervention *n* (%)	Post-Intervention *n* (%)	*p* Value
Occurrence of complications			0.463 ^b^
Yes	72 (62.1)	98 (57.3)	
No	44 (37.9)	73 (42.7)	
Total	116 (100)	171 (100)	
Missing	2	7	
Complications			
Phlebitis	14 (19.2)	35 (35.0)	0.078 ^b^
Infiltration	21 (28.8)	21 (21.0)	0.178 ^b^
Obstruction	23 (31.5)	19 (19.9)	0.060 ^b^
Accidental removal of PIVC	11 (15.1)	14 (14.0)	0.832 ^b^
Others	4 (5.5)	11 (11.0)	0.296 ^b^
Total	73 (100)	100 (100)	
PIVC contamination			0.014 ^b^
Yes	15 (44.1)	10 (17.9)	
No	19 (55.9)	46 (82.1)	
Total	34 (100)	56 (100)	

^b^ Fisher’s exact test.

**Table 4:** Relationship between unequaled variables and PIVC contamination for both study groups.

Unequalled Variables	Pre-Intervention		*p* Value	Post-Intervention		*p* Value
	PIVC Contamination			PIVC Contamination		
	Yes (%)	No (%)		Yes (%)	No (%)	
IV therapeutics			1.000 ^b^			χ^2^ (2) = 0.63
Yes	84.6	85.7		80.0	80.0	*p* = 0.732
No	15.4	14.3		20.0	20.0	
IV antibiotics			1.000 ^b^			0.730 ^b^
Yes	46.2	57.1		30.0	37.5	
No	53.8	42.9		70.0	62.5	
Easiness of puncture	3.1 ± 1.5	3.5 ± 2.1	0.477 ^c^	2.6 ± 1.7	2.5 ± 1.8	0.859 ^c^
Antiseptic			0.314 ^b^			1.000 ^b^
70% Alcohol	66.7	47.4		10.0	10.9	
2% Alcoholic chlorhexidine	33.3	52.6		90.0	89.1	

^b^ Fisher’s exact test, ^c^ Student’s *t*-test.

**Table 5:** Correlation matrix between PIVC contamination and the statistically significant variables for *p* < 0.100 in the analysis (n = 77).

Variables	1	2	3	4	5	6	7
PIVC contamination (1)	1	−0.30 ***	−0.33 ***	0.25 **	0.24 **	0.21 *	0.24 **
70% Alcohol/chlorhexidine (2)		1	0.59 ***	−0.03	−0.04	0.04	0.01
Innovative tourniquet and dressing (3)			1	−0.09	−0.04	−0.05	−0.09
Number of PIVCs during hospital stay (4)				1	0.74 ***	0.17	0.58 ***
Cumulative days with PIVCs (5)					1	0.29 **	0.41 ***
Fluid therapy (6)						1	−0.02
Complications (7)							1

* *p* < 0.10; ** *p* < 0.05; *** *p* < 0.01.

**Table 6:** Binary logistic regression of risk for PIVC contamination.

Independent Variables/Factors	b	SE	X^2^_Wald_ (1)	Sig.	OR	95% CI for OR	
						Lower	Upper
Innovative tourniquet and dressing	−1.55	0.78	3.97	0.046	0.21	0.05	0.98
70% Alcohol/chlorhexidine	−0.84	0.81	1.07	0.302	0.43	0.09	2.12
Complications	1.33	0.64	4.37	0.037	3.80	1.09	13.26
Fluid therapy	1.18	0.62	3.59	0.058	3.26	0.96	11.10
Constant	−0.49						

b: Regression coefficient (b); SE: standard error; X^2^_Wald_ (1): Chi-square Wald test (1 degree of freedom); Sig.: *p*-value; OR: odds ratio; CI: confidence interval.
